# Effect of life stage and pesticide exposure on the gut microbiota of *Aedes albopictus* and *Culex pipiens* L

**DOI:** 10.1038/s41598-020-66452-5

**Published:** 2020-06-11

**Authors:** Elijah O. Juma, Brian F. Allan, Chang-Hyun Kim, Christopher Stone, Christopher Dunlap, Ephantus J. Muturi

**Affiliations:** 1 Department of Entomology, University of Illinois at Urbana-Champaign, 505 S. Goodwin Ave, Urbana, IL 61801 USA; 2 Illinois Natural History Survey, University of Illinois at Urbana-Champaign, 1816 S. Oak St., Champaign, IL 61820 USA; 3 Crop Bioprotection Research Unit, Agricultural Research Service, U.S. Department of Agriculture, 1815 N. University St., Peoria, IL 61604 USA

**Keywords:** Microbial ecology, Microbial ecology, Microbiome, Entomology

## Abstract

Pesticides commonly contaminate the aquatic environments inhabited by mosquito juveniles. However, their role in shaping the mosquito microbiota is not well understood. We hypothesized that environmentally relevant concentrations of atrazine, permethrin and malathion will mediate a shift in the mosquito gut bacterial community structure due to their toxic effect on the aquatic bacterial communities, and reduce mosquito gut bacterial diversity by enriching pesticide-degrading bacterial communities over susceptible taxa. Illumina MiSeq sequencing of the V3-V4 hypervariable regions of the 16 S rRNA gene was used to characterize the microbial communities of larval and adult stages of the two mosquito species and the water samples from microcosms treated with each of the pesticides, separately. Bacterial community composition differed by sample type (larval stage vs. adult stage) and water sampling date (day 3 vs. day 7), but not by pesticide treatment. In larval stages, bacterial OTU richness was highest in samples exposed to malathion, intermediate in permethrin, and lowest in controls. Bacterial richness was significantly higher in larval stages compared to adult stages for all treatments. This study provides a primer for future studies evaluating mosquito microbial responses to exposures to chemical pesticides and the possible implications for mosquito ecology.

## Introduction

One of the central goals of ecology is to understand patterns of species abundance and diversity in communities and ecosystems, and how these patterns are impacted by anthropogenic alterations^1–3^. Pesticides are a common source of environmental disruption, and thousands of different pesticides are used around the world to improve crop production and human health. These pesticides often enter the aquatic habitats, including those that are not intended or legally registered for application to aquatic systems. Consequently, there is considerable research effort to understand and predict the impacts of anthropogenic chemical pesticides on non-target aquatic organisms^4–7^.

Atrazine, permethrin, and malathion are three of the most frequently used pesticides, and their residues are commonly detected in groundwaters of the United States^4,8,9^. Although malathion is still used in agricultural pest control, it is no longer used in vector control in the United States. These three pesticides span a broad range of mode of action and uses. Atrazine, a triazine herbicide, is used globally to control broadleaf weeds by inhibiting electron transport in photosystem II^10^. Ground-water atrazine concentration in the United States range between 0.0002 mg/L-0.05 mg/L with peaks of 1 mg/L reported whenever it rains heavily immediately after field applications^11,12^. Recent monitoring data indicate that the value of 0.053 mg/L represents the 99^th^ percentile of atrazine concentrations in most ground waters of the United States, with a cut-off of 0.1 mg/L used to assess environmental relevance^13^. Atrazine exerts adverse physiological effects on aquatic insects including mosquito larvae at concentrations ranging between 0.02–5 mg/L^12,14^. The effects may include reduced hatching success, lower emergence success, longer emergence times for females, declining species richness, and distorted sex ratio with significant male bias^12,14^. However, exposure to atrazine has also been associated with significantly larger *Aedes albopictus*, a precursor for enhanced vectorial capacity^14^. Soil bacterial taxa, including *Pseduomonas* and *Clavibacter* express *atzA, -B* and -*C* genes that encode enzymes that metabolize atrazine and use the constituents as nitrogen source^15^. Thus atrazine contaminated environments are likely to favor proliferation of bacterial communities that hydrolyze atrazine for energy source^11,16–18^.

Malathion, a broad-spectrum organophosphate, is an inhibitor of acetylcholinesterase that causes acute neurotoxicity to susceptible invertebrates, including insects, at high doses. Malathion is commonly used in agricultural pest and public health vector control in the United States, although its use for public health vector control has declined significantly due to toxicity concerns and resistance development in some mosquito vector species^8,9,19,20^. It is toxic to most aquatic life at concentrations as low as 0.006 mg/L^21^. Reported physiological and behavioral effects of malathion on mosquito life history traits include prolonged development times, reduced interspecific larval competition, toxicity to larvae, and increased vector competence in some species^22^. Malathion concentrations ranging from 0.001 mg/L up to 1 mg/L have been reported in the groundwaters of the United States^8,21–23^.

Permethrin is a neurotoxin that acts by disrupting the functioning of the voltage-gated sodium channels in aquatic invertebrates, including mosquito larvae. In the United States, there has been a steady increase in the usage of pyrethroids in agricultural pest control and public health vector control with permethrin accounting for between 45–60% of all pyrethroids used^24,25^ As a result, it is frequently detected in up to 75% of soils and aquatic sediments, albeit at relatively low concentrations (~10 ng/g dry weight). Permethrin has previously been associated with acute toxicity to several invertebrate indicators of environmental health, including *Chironomus tentans*, and *Hyalella azteca*^24,26^. Studies of its effects on mosquito aquatic stages are limited. However, in one study permethrin was associated with male-biased sex ratio in *Aedes aegypti* mosquitoes^27^. Permethrin concentrations ranging from 0.001–0.002 mg/L have been determined to be effective in routine mosquito control programs^27,28^.

The role of pesticides used in agricultural pest control and public health vector control, in shaping the microbial community composition in the larval environment, and subsequently, the mosquito gut microbial colonization pattern, has received very little attention^29^. Previous studies have mostly investigated biotic endpoints, including microbial productivity, enzymatic activity, biomass, and respiration^30–32^. The lone study that applied next-generation sequencing to evaluate the effect of pesticides on microbial community composition focused on the larval environment, but not the mosquito gut bacterial communities^29^.

In this study, we used an experimental microcosm approach to evaluate whether environmentally relevant concentrations of atrazine, permethrin, and malathion in mosquito larval habitats play an important role in shaping the bacterial communities of mosquitoes and their larval environment. We used *Culex pipiens* L. and *Aedes albopictus*, two container-dwelling mosquitoes, as the model species for this study^33–36^. The two species thrive in diverse aquatic habitats embeded within agricultural landscapes, and thus are likely to be exposed to pesticides from agricultural applications or public health vector control initiatives^37^. *Culex pipiens* L. is an introduced European species that arrived in North America in the early 16^th^ Century through trade, and has been naturalized in the United States north of 39 ° latitude^38–40^. It serves as both an amplifying and bridge vector for West Nile virus and St. Louis encephalitis due to its preference for feeding on birds^41^. *Aedes albopictus* is an invasive species first reported in the United States in August 1985 in Harris County, Texas, and has so far been reported in over 27 states^36,42,43^. It is a known vector of dengue and Chikungunya viruses^44^, but experimental studies indicate that it can be a competent vector of up to 32 other viruses, 13 of which are present in the United States^36^.

We used Illumina MiSeq sequencing of the V3-V4 hypervariable regions of the bacterial 16 S rRNA gene to determine how environmentally relevant concentrations of atrazine, malathion and permethrin affect the microbial communities of the two mosquito species and their larval environment. We hypothesized that: 1). Environmentally relevant concentrations of atrazine, permethrin and malathion will mediate a shift in the mosquito gut microbial community structure due to their toxic effect on the aquatic microbial communities; 2). Environmentally relevant concentrations of atrazine, permethrin and malathion will reduce mosquito gut microbial diversity by promoting the growth of microbial taxa that use pesticides as a source of carbon while eliminating pesticide-susceptible bacterial taxa. This study provides a basis for future studies investigating the responses of bacterial communities in the larval environment and the mosquito gut to exposures to chemical pesticides varied at different concentrations and the potential implications on mosquito ecology.

## Results

### Sequence processing and alpha diversity

Illumina sequencing of the V3-V4 hypervariable region of the 16 S rRNA gene amplicons yielded a total of 7,147,541 raw sequence reads from 560 samples (200 *Ae. albopictus*; 200 *Cx. pipiens* L.; 160 water samples). After removing chimeric and other non-bacterial sequences, and quality filtering to remove bacterial OTUs constituting <0.005% of the total sequences and rarefying to an even depth of 1002 sequences per sample to standardize sample size, a total of 466 samples (138 *Ae. albopictus*; 182 *Cx. pipiens* L.; 146 water samples) were retained. The final sample size consisted of a total of 7,064,485 sequences (mean ± SE 15,733.82 ± 2933.51; min = 358; max = 944,292) that were clustered into 449 bacterial OTUs at 97% sequence similarity, which is adequate for bacterial identification to the genus level.

### Microbial communities associated with *Aedes albopictus*

Rarefaction curve analysis (an interpolation and extrapolation method for comparing species richness based on samples of equal sizes)^45,46^ indicated that most OTUs were recovered by the sequencing depth coverage of 1002 sequences. Chao1 estimator, an abundance-based diversity index for estimating rare species, revealed that up to 80.7% ± 0.01 (mean ± SE) of the OTUs were recovered. Bacterial OTU richness was highest in larval samples exposed to permethrin, and lowest in adult samples exposed to permethrin (Fig. S1). The observed OTUs, expected OTU richness (Chao1), and OTU diversity were significantly higher in larval samples compared to adult samples (Observed OTUs: Kruskal-Wallis chi-squared = 153.34, df = 15, *p* < 0.0001; Chao1: Kruskal-Wallis chi-squared = 156.15, df = 15, *p* < 0.0001; Shannon index: Kruskal-Wallis chi-squared = 172.14, df = 15, *p* < 0.0001) (Fig. S2A).

### Taxonomic classification and bacterial composition across pesticide treatments

**Figure 1:**
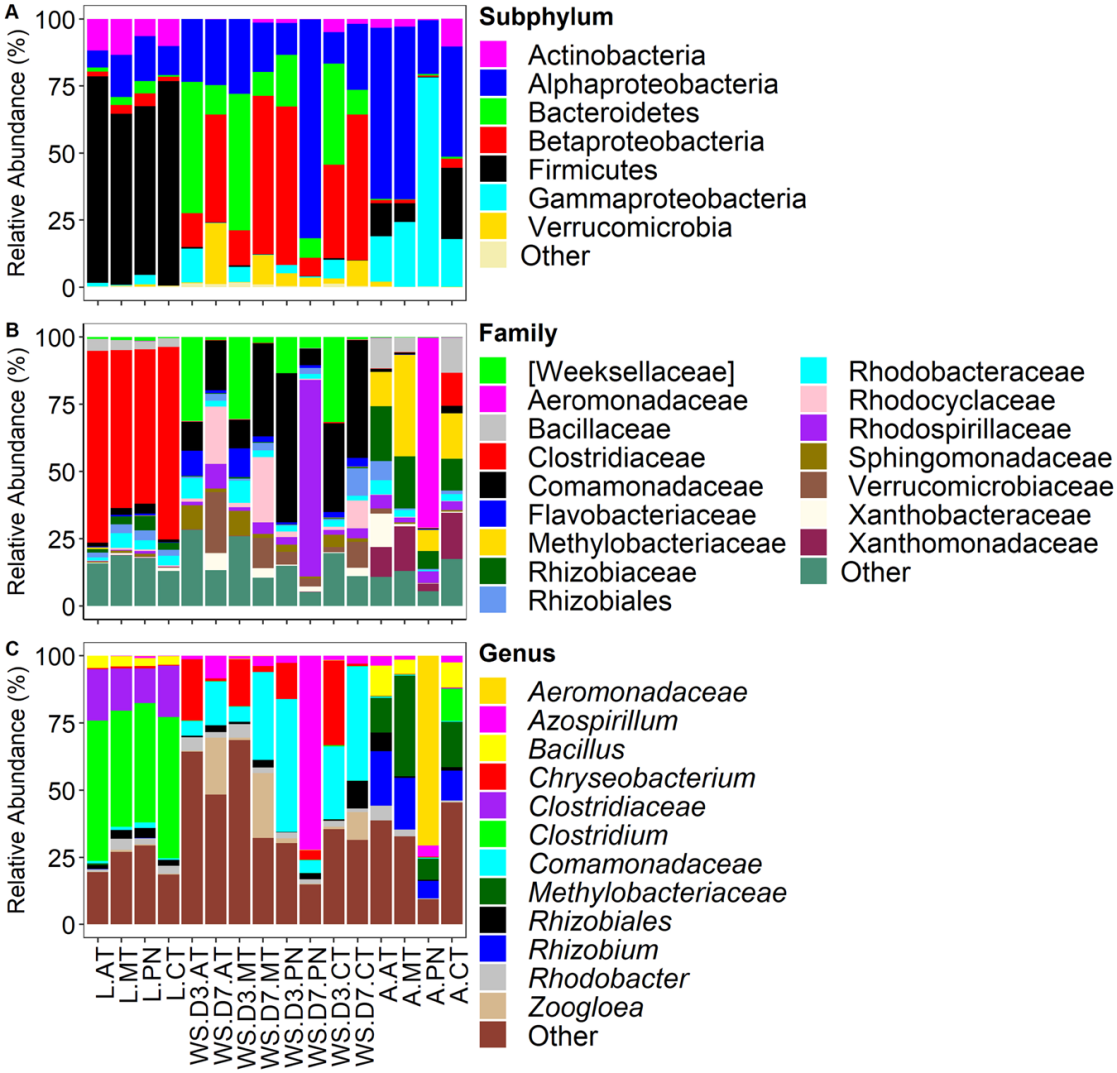
Composition of bacterial communities in *Ae. albopictus* and water samples. Taxa with sequence abundance <1% of total sequences were pooled together as “Other” in all the taxonomic ranks. Panel A – relative abundance at subphylum level; panel B – relative abundance at family level; panel C – relative abundance at genus level. L – Larvae; A – Adults; AT – Atrazine; MT – Malathion; PN – Permethrin; CT – Control; WS – Water Sample; D3 – Day 3; D7 – Day 7. Figures were generated using R version 3.6.1^76^ (https://www.r-project.org/) within the RStudio environment version 1.2.1335^77^ (https://rstudio.com/).

To ascertain relative abundance of the bacterial taxa at each taxonomic level the 449 bacterial OTUs were classified into seven phyla, 20 classes, 72 families, and 104 genera. The most dominant phyla were Proteobacteria (51.4%), Firmicutes (28.3%), and Bacteroidetes (12.1%), and the rest were <10%, cumulatively (Fig. 1A). At the family level, Clostridiaceae (25.8%) Comamonadaceae (13.9%) and Rhodospirillaceae (10.3%) were dominant while the rest were <10%, cumulatively (Fig. 1B). At the genus level, *Clostridium* (19.2%), unclassified Comamonadaceae (11.7%) and *Azospirillum* (10.0%) were dominant while the rest were <10%, cumulatively (Fig. 1C). Bacterial OTUs classified as *Wolbachia* were dominant overall at the genus level but were excluded from the analysis because *Wolbachia* is a maternally inherited obligate intracellular microbial symbiont naturally occurring in *Ae. albopictus*^47^, therefore it was not considered a gut bacteria for the purposes of the analyses. Overall, 183 (43.9%) OTUs were shared among larvae, adults, and water samples from all pesticide treatments. Twenty-five, 14, and 11 bacterial OTUs were unique to larval, adult, and water samples, respectively (Fig. S4A). Three hundred and seventy-one bacterial OTUs were detected in larval samples compared to 253 in adult samples, and 324 and 320 in day three water samples (WS.D3) and day seven water samples (WS.D7), respectively. The differences in OTUs detected per sample were statistically significant (Chi-squared = 22.3, df = 3, *p* < 0.0001). Multiple pairwise comparison with Bonferroni corrections revealed three statistically different sample groups (larvae vs. adults, *p* < 0.0001; adult vs. WS.D7, *p* = 0.019; adult vs. WS.D7, *p* = 0.031).

Non-metric multidimensional scaling (NMDS) analysis using Bray-Curtis distance matrix revealed three clusters separating the microbial community composition by sample type (larvae, adult or water samples), and water sampling date (D3 vs. D7) but not by pesticide treatment (Fig. 3). These differences were confirmed by statistically significant pairwise comparisons using multi-response permutation procedure (MRPP) analysis with Bonferroni-adjusted p-values (Table 1). This is a non-parametric test for testing the hypothesis of no difference between two or more groups. Non-parametric multivariate analysis was performed to test for the effect of pesticide treatment and the day of water sampling on the water chemistry parameters. There were significant variations temperature, dissolved oxygen, conductivity, and total dissolved oxygen, for day 3 and 7 water samples but varied by pesticide treatment (Fig. S5, Table S1).

**Figure 2:**
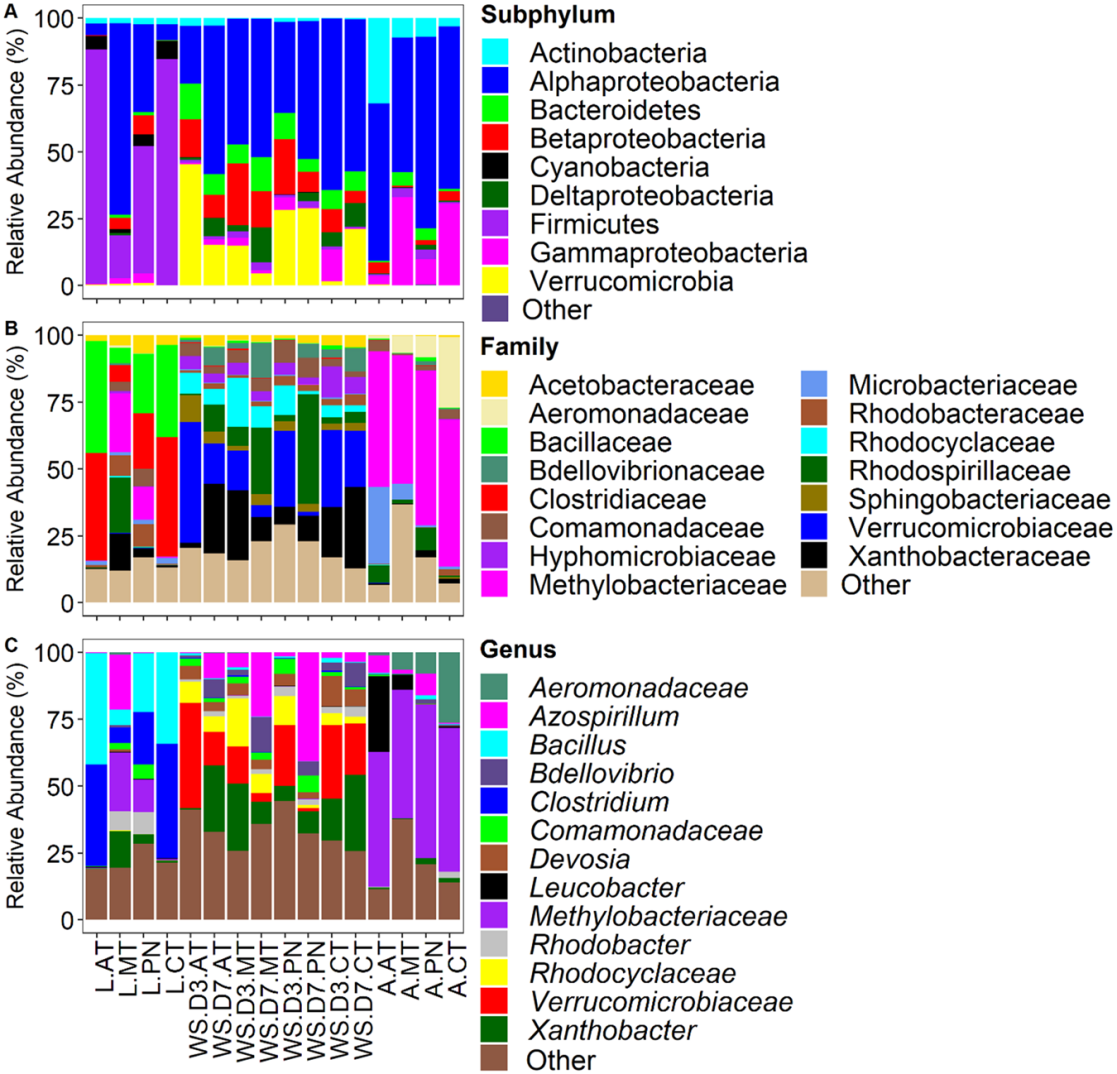
Composition of bacterial communities in *Cx. pipiens* L. and water samples. Taxa with sequence abundance <1% of total sequences were pooled together as “Other” in all the taxonomic ranks. Panel A – relative abundance at subphylum level; panel B – relative abundance at family level; panel C – relative abundance at genus level. L – Larvae; A – Adults; AT – Atrazine; MT – Malathion; PN – Permethrin; CT – Control; WS – Water Sample; D3 – Day 3; D7 – Day 7. Figures were generated using R version 3.6.1^76^ (https://www.r-project.org/) within the RStudio environment version 1.2.1335^77^ (https://rstudio.com/).

**Figure 3:**
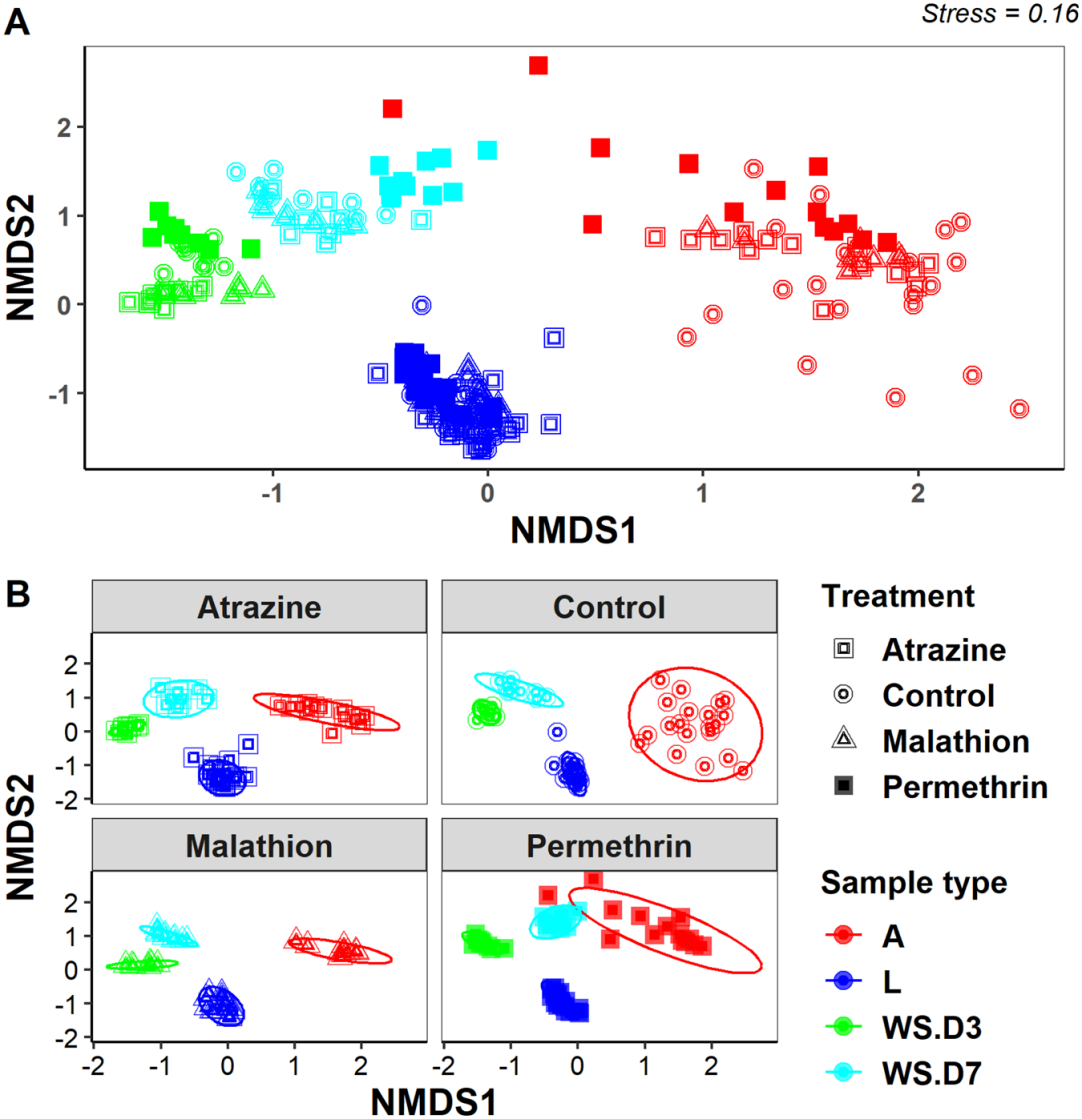
NMDS based on Bray-Curtis distance ordination of bacterial communities from *Ae. albopictus* larval, adult, and water samples from pesticide treatments and control group. (**A**) – all mosquito and water samples presented together; (**B**) – mosquito and water samples partitioned by the treatment or control from which they were obtained. L – Larvae; A – Adults; WS – water samples; D3 – Day 3; D7 – Day 7. Figures were generated using R version 3.6.1^76^ (https://www.r-project.org/) within the RStudio environment version 1.2.1335^77^ (https://rstudio.com/).

**Table 1:** MRPP results showing differences in bacterial communities of *Ae. albopictus* mosquitoes by life stage and water sample type.

Sample type			Ad			N			T	A	*P*
_L_	_vs._	_A_	_0.12_	_vs._	_0.26_	_81_	_vs._	_57_	_−83.75_	_0.44_	_<0.001_
_L_	_vs_	_WS.D3_	_0.12_	_vs._	_0.17_	_81_	_vs._	_36_	_−67.25_	_0.38_	_<0.001_
_L_	_vs_	_WS.D7_	_0.12_	_vs._	_0.21_	_81_	_vs._	_37_	_−68.48_	_0.38_	_<0.001_
_A_	_vs_	_WS.D3_	_0.26_	_vs._	_0.17_	_57_	_vs._	_36_	_−56.54_	_0.46_	_<0.001_
_A_	_vs_	_WS.D7_	_0.26_	_vs._	_0.21_	_57_	_vs._	_37_	_−55.21_	_0.44_	_<0.001_
_WS.D3_	_vs_	_WS.D7_	_0.17_	_vs._	_0.21_	_36_	_vs._	_37_	_−30.50_	_0.29_	_<0.001_

Bacterial communities differed significantly between groups (larvae vs. adult samples vs. water samples). L – Larvae; A – Adults; WS – Water Sample; D3 – Day 3; D7 – Day 7.

Ad average within-group distances for mosquito species

*N* sample size

*T*-test statistic describing separation within groups.

*A* chance-corrected within-group agreement as log_10_.

### Microbial communities associated with *Culex pipiens L*

Rarefaction curve analysis recovered most of the OTUs by the sequencing depth coverage of 1002 sequences. Chao1 estimator analysis detected up to 86.0% ± 0.01 (mean ± SE) of the expected bacterial OTUs. Bacterial OTU richness was highest in day three water samples exposed to permethrin, and lowest in adult samples exposed to malathion (Fig. S3). The observed OTUs, expected OTU richness (Chao1), and OTU diversity were significantly higher in larval samples compared to adult samples (Observed OTUs: Kruskal-Wallis chi-squared = 176.77, df = 15, *p* < 0.0001; Chao1: Kruskal-Wallis chi-squared = 186.53, df = 15, *p* < 0.0001; Shannon index: Kruskal-Wallis chi-squared = 165.47, df = 15, *p* < 0.0001) (Fig. S2B).

### Taxonomic classification and bacterial composition across pesticide treatments

The most dominant phyla were Proteobacteria (60.5%) and Firmicutes (25.7%), the rest were <10%, cumulatively (Fig. 2A). At the family level Methylobacteriaceae (26.7%), Clostridiaceae (11.85%) and Bacillaceae (10.96%), were dominant, the rest were <10%, cumulatively (Fig. 2B). At the genus level the unclassified Methylobacteriaceae (26.5%), *Clostridium* (11.03%), *Bacillus* (10.58%), were dominant while the rest were <10%, cumulatively (Fig. 2C). Overall, 258 (58.6%) OTUs were shared among larvae, adults, and water samples from all pesticide treatments (Fig. S4B). Four-hundred and six bacterial OTUs were detected in larval samples compared to 394 in adult samples, and 329 and 335 in WS.D3 and WS.D7, respectively. The differences in OTUs detected per sample were statistically significant (Chi-squared = 12.9, df = 3, *p* < 0.005). Multiple pairwise comparison with Bonferroni corrections revealed only one statistically different sample group (larvae vs. WS.D3, *p* = 0.027).

**Figure 4:**
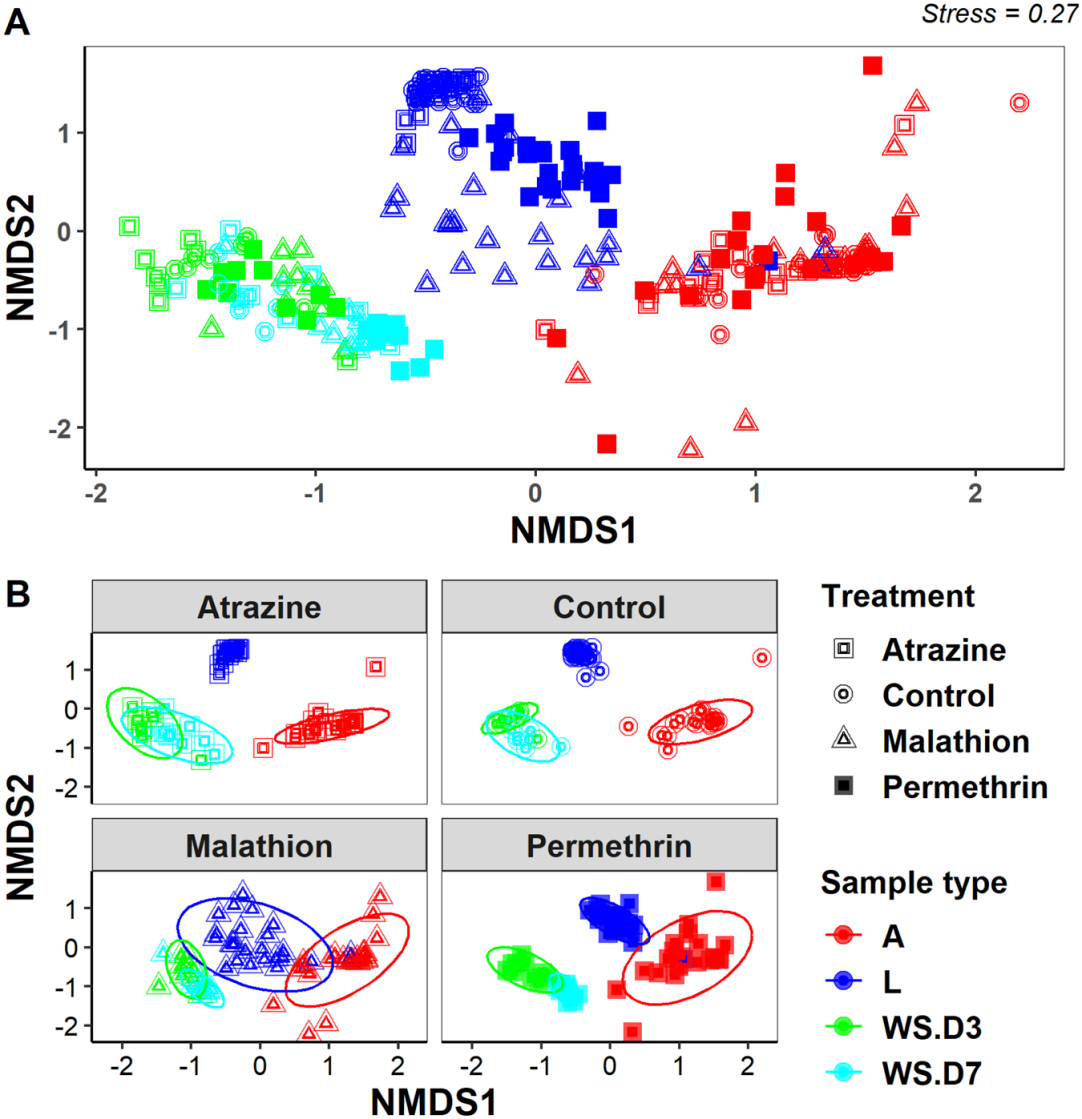
NMDS based on Bray-Curtis distance ordination of bacterial communities from *Cx. pipiens* L. larval, adult, and water samples from pesticide treatments and control group. (**A**) – all mosquito and water samples presented together; (**B**) – mosquito and water samples partitioned by the treatment or control from which they were obtained. L – larvae; A – Adult; WS – water samples; D3 – Day 3; D7 – Day 7. Figures were generated using R version 3.6.1^76^ (https://www.r-project.org/) within the RStudio environment version 1.2.1335^77^ (https://rstudio.com/).

Non-metric multidimensional scaling (NMDS) analysis revealed three clusters separating the microbial community composition by sample type (larvae, adult or water samples), and water sampling date (D3 vs. D7) but not by pesticide treatment (Fig. 4). These differences were confirmed by statistically significant pairwise comparisons using MRPP analysis with Bonferroni-adjusted p-values (Table 2). There were significant variations in all the physical chemical parameters evaluated for day 3 and 7 water samples but varied by pesticide treatments (Fig. S6, Table S2).

**Table 2:** MRPP results showing differences in bacterial communities of *Cx. pipiens* L. mosquitoes by life stage and water sample type.

Sample type			Ad			N			T	A	*P*
_WS.D3_	_vs._	_WS.D7_	_0.22_	_vs._	_0.22_	_36_	_vs._	_37_	_−11.60_	_0.12_	_<0.001_
_WS.D3_	_vs._	_L_	_0.22_	_vs._	_0.21_	_36_	_vs._	_94_	_−66.76_	_0.34_	_<0.001_
_WS.D3_	_vs._	_A_	_0.22_	_vs._	_0.28_	_36_	_vs._	_88_	_−69.51_	_0.37_	_<0.001_
_WS.D7_	_vs._	_L_	_0.22_	_vs._	_0.21_	_37_	_vs._	_94_	_−63.07_	_0.32_	_<0.001_
_WS.D7_	_vs._	_A_	_0.22_	_vs._	_0.28_	_37_	_vs._	_88_	_−64.74_	_0.35_	_<0.001_
_L_	_vs._	_A_	_0.21_	_vs._	_0.28_	_94_	_vs._	_88_	_−85.64_	_0.33_	_<0.001_

Bacterial communities differed significantly between groups (larvae vs. adult vs. water samples). L – Larvae; A – Adults; WS – Water Sample; D3 – Day 3; D7 – Day 7

Ad average within-group distances for mosquito species

*N* sample size

*T*-test statistic describing separation within groups

*A* chance-corrected within-group agreement as log_10_.

## Discussion

This study evaluated the effect of exposure to environmentally relevant concentrations of three pesticides, atrazine, permethrin, and malathion, on the diversity and composition of the gut bacterial communities of two container-dwelling mosquitoes, *Ae. albopictus* and *Cx. pipiens* L. Unexpectedly, malathion and permethrin treatments were associated with higher bacterial richness in larval mosquito samples and some water samples, for both *Ae. albopictus* and *Cx. pipiens* L. The lowest bacterial richness was reported in adult mosquito samples regardless of the pesticide treatment. Our study findings did not support our initial hypothesis that exposure to environmentally relevant pesticide concentrations could also induce a shift in the mosquito gut microbial community structure. However, there was evidence of distinct clustering of bacterial OTUs based on the mosquito life stage (larvae vs. adults) and the date in which water samples were collected from the microcosms (D3 vs. D7).

We report significantly higher bacterial richness associated with malathion and permethrin treatments in both larval samples and water samples, contrary to our initial hypothesis that exposure to environmentally relevant pesticide concentrations will reduce mosquito gut microbial diversity. Bacterial communities vary in their sensitivity to chemical pesticides^48^. Malathion in the natural environment has a short half-life ranging from a few hours to 1 week. It is broken down by several bacterial taxa into ∝ and β-malathion monoacid, malathion dicarboxylic acid, O,O-dimethyl phosphorodithioic acid, and diethyl maleate^19,21^. Permethrin is degraded to 3-phenoxybealdehyde and 3-phenoxybenzoic acid in the natural environment. Unlike malathion, permethrin is more stable in the environment than malathion and has the potential to exert its toxic effect over a longer period. However, the effect of permethrin may be diminished by its tendency to adsorb to dissolved solids or sediment surfaces and thus reduce the bioavailable concentrations able to induce bacterial toxicity^24,49^. We speculate that the increase in bacterial diversity in permethrin treatments may be the result of permethrin favoring enrichment of more bacterial species that degrade it or by eliminating dominant taxa that inhibit the growth of rare species communities^30,32,50^. Even though the concentrations of permethrin and malathion we used induced mortality in the mosquito larvae, it is likely that the fraction that was bioavailable to act on bacterial communities in the microcosms and in the mosquito guts was too low to induce significant shift in the bacterial richness and composition. Due to the low bioavailable amount, it is possible that the bacterial communities recovered rapidly from the initial short-term pulse of pesticide exposure.

Exposure of bacterial communities to pesticides in the environment produce variable effects that may be dependent on many factors, including the pesticide concentrations used, pesticide type, the physical and chemical characteristics of the study environment, and the study system (e.g., lake, estuary, or microcosm)^29,31,50^. Under certain conditions pesticides may promote bacterial diversity in the aquatic environments, partly due to the pesticides eliminating protozoa that graze on bacteria, thus releasing the bacterial communities from grazing pressure^48,51^. We suspect that this phenomenon may have been at play in the permethrin and malathion treatments hence the higher bacterial diversity observed. Widenfal *et al*. (2008), using two different concentrations (“low” – 150 µg/kg, and “higher” −1000 times higher than the low concentrations), showed a lack of direct correlation between pesticide concentration and shifts in bacterial community composition. For instance, they reported shifts in bacterial community composition with captan and glyphosate at “low” concentrations but not “high” concentrations, possibly indicating that low concentrations possibly stimulate bacterial taxa that use pesticide as carbon source, or the low pesticide concentration eliminate susceptible taxa thus releasing the tolerant bacterial taxa from competition. When the experiment was monitored over a 30-day duration, there was clustering of bacterial communities between samples exposed to low vs. high concentrations from the different chemical pesticides tested. In this present study, only one pesticide concentration was applied per treatment, which may not be effective for monitoring shifts in bacterial community structure over time. Mosquitoes aquatic stages may be exposed to repeated short-term pulses of pesticides of varying concentrations during peak agricultural season or vector control initiatives. As such, additional studies with repeated pesticide exposures over longer periods will be helpful in decoupling bacterial communities’ responses to pesticide exposure in the mosquito guts and the larval environment.

Some of the bacterial taxa regularly isolated in the mosquito gut, including *Bacillus*, *Aeromonas, Flavobacterium*, *Xanthomonas* sp. and *Pseudomonas* sp, have been implicated in the biodegradation of malathion and permethrin insecticides in the natural environment^19,21,25,49,52,53^. Our study isolated *Flavobacterium, Pseudomonas* and *Bacillus* water samples and the mosquito samples. In *Ae. albopictus* experiments, *Bacillus* occurred in small proportions in larval samples, while *Flavobacterium* was present in small fraction in water samples regardless of the pesticide treatment. Whereas these bacterial taxa have been associated with malathion and permethrin degradation, evidence from our study does not point to them exploiting this characteristic to bias their own proliferation at the expense of permethrin- or malathion-sensitive taxa. This further explains the lack of reported reduction in bacterial richness in the microcosm or in the mosquito gut microbiota that would be attributable to proliferation of some bacterial taxa over others due to the effect of the pesticides.

We observed shifts in the bacterial community composition between larval and adult mosquito samples. Similarly, significantly higher bacterial diversity was observed in larval stages compared to adult stages, in line with previous studies^54–58^. It is well established that mosquitoes acquire a substantial fraction of their microbial commensals from their environment as larvae, or through imbibing of the larval water at metamorphosis^59–63^. During metamorphosis a portion of the mosquito microbial symbionts may be shed or sequestered due to modifications to the meconial peritrophic membrane; others may be lost through digestion and excretion, thus rendering them undetectable^64–66^. This provides a possible explanation to the observed shift in the microbial diversity and composition between the larval and adult mosquitoes. In this study, the adult mosquito samples were preserved immediately post-emergence. Thus, their microbial assemblages could only have been acquired transstadially, vertically, or by imbibing of the larval water at emergence^64^. The physiological states in the midguts of larvae, compared to adult mosquitoes, such as pH, redox conditions, digestion, assimilation, and excretion rates, also potentially support the proliferation of distinct microbial assemblages^60,67,68^. This could provide an additional source of variation in the microbial composition and diversity between larvae and adult mosquitoes.

Microcosm studies provide an ideal starting point for understanding interactions between organisms and their environment since the experimental conditions can be carefully controlled. In the natural environment, factors such as nutrient pollutants from fertilizer residues can alter the aquatic microbial community response to pesticides by either stimulating or inhibiting the action of pesticides on the microbial communities^48,69^. Additionally, the interaction among a suite of pesticides in the natural environment is common and may also have similar stimulatory or inhibitory effects. Our experimental design evaluated each pesticide individually, yet sometimes significant disruptions of microbial communities may only be possible where a mixture of pesticides is involved as could be the case in the natural environment. Evidence from previous studies have also shown that factors such as adsorption of pesticides to dissolved solids in the aquatic environment affect the bioavailable amount that can induce shifts in aquatic or soil bacterial communities. Additionally, in the natural environment, pesticides undergo photolytic breakdown, resulting in intermediate compounds that affect the microorganisms differently compared to the parent compound^15,30,48,70^. However, we did not evaluate this in our study. The interpretation of our experiment is also limited by the fact that the experiment only characterized the microbial communities of the mosquito samples that survived the pesticide treatments. Additional future characterization of the microbial communities of the dead and surviving sample treatments is likely to be informative in helping to disentangle the selective effect of chemical pesticides on microbial community composition and diversity. Such studies should also be done across a broader range of pesticide concentrations to capture the full range of lethal and sublethal outcomes.

In summary, we have shown that environmentally relevant concentrations of permethrin and malathion are associated with higher bacterial diversity in larval mosquito samples, but no detectable shift in the mosquito microbial community composition was reported for any of the pesticides evaluated. The effect of pesticides on the microbial community composition and diversity in the aquatic environments, and subsequently in the mosquito guts, can be complex and difficult to predict and may vary significantly depending on pesticide type, concentrations used and even the system being studied^29,30,48^. Similar studies in the future using both culture-independent and culture-dependent methods should vary pesticide concentrations and stagger the application to account for pesticide degradation and disappearance in the study system. This study forms a basis for future studies evaluating the effect of varied pesticide concentrations on the larval environment, and the mosquito gut bacterial communities and the potential implications on mosquito biology and ecology.

## Materials and Methods

### Mosquito sampling and colony establishment

Oviposition traps (ovitraps) were established in ten selected sites within residential neighborhoods in Champaign County, IL, for the sampling of *Cx. pipiens* and *Ae. albopictus* eggs. Three ovitraps were established at each of the ten selected sites for a total of 30 ovitraps. The traps consisted of 19 L white cylindrical buckets with five 80 mm-diameter overflow holes 18 cm from the base. Each ovitraps was baited with three liters of 3-day old grass infusion prepared following the method described in Jackson *et al*.^71^. To sample *Ae. albopictus* eggs, each ovitrap was lined with strips of germination paper and placed in shaded areas under trees or shrubs. Sampling was conducted twice every week between July 3 and September 20, 2017. The germination papers containing the sampled eggs were individually hatched in media consisting of 700 ml of warm (25 ± 1 °C) deionized water, 0.25 g of CM0001 Nutrient Broth (Oxoid, Hampshire, England) and 0.05 g yeast in enamel pans^72^. Emerging adult mosquitoes were identified to species using morphological characteristics and *Ae. albopictus*, the most abundant *Aedes* species in the samples, was selected for this experiment. The adults were pooled in a paperboard cage and artificially blood fed on citrated bovine blood. The resulting eggs were hatched and used for the microcosm experiment.

*Culex pipiens* egg rafts were sampled concurrently using the same ovitraps used for *Ae. albopictus* sampling. Egg rafts were collected using a paintbrush and placed on moist filter papers in a petri dish and transported to the laboratory. In the laboratory, egg rafts were hatched separately in Petri dishes and first instar larvae of *Cx. pipiens* L. were distinguished from *Cx. restuans* (approx. 12–15 hours old) based on the presence of a clear scale anterior to the sclerotized egg-breaker in *Cx. restuans* but not in *Cx. pipiens* L^39^. First instar larvae of the identified *Culex pipiens* L. were pooled regardless of the ovitrap origin and used in the microcosm experiment.

### Microcosm experiment

A completely randomized experimental design was used, consisting of four pesticide treatments (atrazine, malathion, permethrin, control). Two sets of experiments were established for each mosquito species. In the first set, first instar larvae were introduced to each microcosm and their development monitored up to late 4^th^ instar larvae after which a minimum of 15 larval samples surviving from each treatment were preserved at −80 °C for larval microbiome analysis. In the second set, first instar larvae were added to each microcosm and reared to adulthood. Newly emerging adults were preserved at −80 °C for adult microbiome analysis. Each treatment was replicated 5 times for a total of 80 containers. For each mosquito species, 50 first instar larvae were randomly assigned to each replicate. The microcosms consisted of 1 L of grass infusion in 5 L cylindrical containers. Each pesticide was diluted in acetone to make stock solutions. In the *Cx. pipiens* L. study, on day 0, each microcosm was treated with the requisite concentration of the stock solution of each target pesticide diluted in 500 µL acetone to make a final concentration of 5 mg/L atrazine, 0.05 mg/L malathion and 0.03 mg/L permethrin. Larval mortality was monitored over a 24-hour period. Since no visible larval mortality was observed after the first 24 hours, an equal concentration of malathion and permethrin in 500 µL of acetone were added to their respective treatments to make a final concentration of 0.1 mg/L malathion, and 0.06 mg/L permethrin, respectively. The average larval mortality by pesticide treatment in the microcosms ranged between 6–69.2% (Fig. S7A). Similarly, in microcosm experiments for *Ae. albopictus*, after 24 hours when no mortality was observed, an equal concentration of malathion in 500 µL of acetone was added to make a final concentration of 0.1 mg/L malathion. The average larval mortality by pesticide treatment in the microcosms ranged between 11.6–56.8% (Fig. S7B). For each control experiment, an equivalent volume of 500 µL acetone was added to each control microcosm. The atrazine and malathion concentrations used in this experiment were based on concentrations previously reported from the environment^16,73,74^. The concentrations used for permethrin were determined by conducting preliminary bioassays with both *Cx. pipiens* L. and *Ae. albopictus* to determine LC_50_ values. The LC_50_ values obtained in our bioassays are similar to those from previous studies that have been found to induce acute toxicity in mosquito larval stages^27,28^.

Microcosms were maintained under controlled conditions of 27 °C ± 2 and ~75 ± 5% relative humidity. In the adult mosquito experimental component, emergence data were recorded, and at least ten newly emerged females were collected per replicate and preserved at −80 °C for later microbial DNA analysis. In addition, aliquots of 30 mL grass infusion samples were collected from all microcosms on days three and seven and preserved at −80 °C to compare the microbiota of the mosquito guts to the aquatic environment in the microcosm. Physiochemical parameters of the microcosms, including pH, temperature, conductivity, salinity, total dissolved solids, and dissolved oxygen, were taken using ExStik meters (Extech Instruments, Nashua, NH).

### DNA extraction, 16 S rRNA gene library preparation and sequencing

Frozen mosquito samples were surface-sterilized in 70% ethanol solution for 5 minutes, transferred to 3% bleach solution for 3 minutes, and then once more in 70% ethanol for another 5 minutes. Samples were then rinsed five times in sterile water and once in 0.8% saline solution to rid them of any alcohol^56^. Dissections were carried out under a stereomicroscope. Each gut sample was dissected in a drop of physiological saline solution on a sterile microscope slide and used for DNA extraction. Fifty mosquito samples were used per treatment: 25 larval samples and 25 adult samples divided evenly among each treatment replicate. Water samples were thawed, and the total volume of 30 mL centrifuged at 5000 rpm for 20 mins at 3 °C (Centrifuge 5810 R, Eppendorf, Hamburg, Germany), and microbial DNA extracted from each sample pellet. Midgut samples and water samples were individually suspended in bead solution of PowerSoil DNA Isolation Kit, homogenized using Retsch MM 300 TissueLyser (Retsch, Haan, Germany) and genomic DNA extracted using DNeasy PowerLyzer PowerSoil DNA Isolation Kit (Qiagen Inc., Valencia, CA) according to the manufacturer’s instructions. DNA was quantified using Nanodrop 1000 (ThermoFisher Scientific, Pittsburgh, PA). The V3-V4 hypervariable region of the bacterial 16 S rRNA gene was PCR-amplified using the following primer set specific for V3-V4 region of the 16 S rRNA gene: Forward 5′CCTACGGGNGGCWGCAG; Reverse 5′GACTACHVGGGTATCTAATCC. PCR was done in 25 µL reactions containing 12.5 µL of 2x KAPA HiFi HotStart ReadyMix, 5 µL of 1 µM each of the forward and reverse primers, and 2.5 µL of template genomic DNA. PCR conditions were 95 °C for 3 min; 25 cycles of: 95 °C for 30 s, 55 °C for 30 s, 72 °C for 30 s; 72 °C for 5 mins; hold at 4 °C. PCR amplicons were cleaned using AMPure XP beads to remove free primers and primer-dimer species, and a second PCR was conducted using Nextera XT Index Kit (Illumina, San Diego, CA) to attach dual indices and Illumina sequencing adapters^72^. Index PCR was conducted in 45 µL reactions containing 25 µL of 2x KAPA HiFi HotStart ReadyMix, 5 µL each of index 1 and index 2 combinations, and 10 µL of PCR grade water. Thermocycling conditions were 95 °C for 3 min; 8 cycles of 95 °C for 30 s, 55 °C for 30 s, 72 °C for 30 s; 72 °C for 5 mins; hold at 4 °C. A negative control sample made up of DNA extracted from molecular biology grade water was sequenced with the same protocol to allow detection of the contamination background. PCR amplicons were cleaned and normalized using a SequalPrep normalization plate (Thermofisher Scientific, Waltham, MA). The pooled library was mixed with Phix control spike-in of 5% as a sequencing control. The samples were sequenced using an Illumina MiSeq system with a MiSeq V3 2 × 300 bp sequencing kit. The demultiplexed reads were quality-trimmed to Q30 using CLC genomics workbench v8.5 (Qiagen Inc., Valencia, CA). Read pairing, fixed-length trimming and OTU clustering were done using CLC Bio Microbial Genomics module (Qiagen Inc., Valencia, CA) using the reference sequences from the Greengenes ribosomal RNA gene database^75^.

### Statistical analysis

All analyses were conducted using R version 3.6.1^76^ within the RStudio environment version 1.2.1335^77^, and PC-ORD version 6.08^78^ statistical packages. Bacterial OTUs consisting of <0.005% of the total sequences were removed prior to analysis to eliminate artifacts of PCR and sequencing^79^. Sequences were rarefied to a depth of 1002 reads per sample to correct for unequal sample sizes. From a starting sample size of 560 samples, 94 samples had <1002 reads and were removed from further analysis. Rarefaction curves were fitted to estimate sample coverage^80,81^ using the “phyloseq” package version 1.28.0 in R^82^. Rarefaction curves were generated using unrarefied data at a maximum read depth of 1002 sequences. Alpha diversity metrics, including Shannon diversity index, observed species, and chao1 estimator were generated in QIIME 2^83^, and their means and 95% confidence intervals calculated to test for significant differences in alpha diversity indices between pesticide treatments. Kruskal-Wallis test was used to test for differences in the means between sample types and pairwise Wilcoxon rank-sum test with Bonferroni correction performed to separate significant pesticide treatments. Beta-diversity measures were computed using the Bray-Curtis dissimilarity index using the “phyloseq” package and non-metric multidimensional scaling (NMDS) ordination plots generated to visualize the results. A multi-response permutation procedure (MRPP) was conducted in PC-ORD version 6.08^78^ to determine significant differences in microbial communities between pesticide treatments. The p-values were Bonferroni-corrected to reduce the chances of type I error. Non-parametric multivariate analysis was performed to test for the effect of pesticide treatment and the day of water sampling on the water chemistry parameters, using the function “nonpartest” from the R package “npmv” version 2.4.0. Significant treatment factors across water chemistry parameters were delineated using the function “ssnonpartest”^84^. Venn diagrams were constructed using the R package “limma”^85^ version 3.40.2 to visualize OTUs that were shared between larvae, adults and water samples for both *Cx. pipiens* L. and *Ae. albopictus* samples.

## Supplementary information

Supplementary information.
