# Supplementary material for: Effect of life stage and pesticide exposure on the gut microbiota of Aedes albopictus and Culex pipiens L
Source: Sci Rep. 2020 Jun 11;10:9489. doi: 10.1038/s41598-020-66452-5 (PMC7289809; doi:10.1038/s41598-020-66452-5)
Supplement: Supplementary file 1 — Supplementary information. [file 41598_2020_66452_MOESM1_ESM.pdf]

Effect of life stage and sublethal pesticide exposure on the gut microbiota of *Aedes albopictus* and *Culex pipiens* L.

Elijah O. Juma[1*], Brian F. Allan[1], Chang-Hyun Kim[2], Christopher Stone[2], Christopher Dunlap[3], Ephantus J. Muturi[3]

[1]Department of Entomology, University of Illinois at Urbana-Champaign, 505 S. Goodwin Ave, Urbana, IL 61801, USA

[2]Illinois Natural History Survey, University of Illinois at Urbana-Champaign, 1816 S. Oak St., Champaign, IL 61820, USA

[3]Crop Bioprotection Research Unit, Agricultural Research Service, U.S. Department of Agriculture, 1815 N. University St. Peoria IL 61604, USA

*Corresponding Author: 505 S. Goodwin Ave, Urbana, IL 61801, USA. Tel: +1 217 979 9127; E-mail: elijahjuma@gmail.com; juma2@illinois.edu

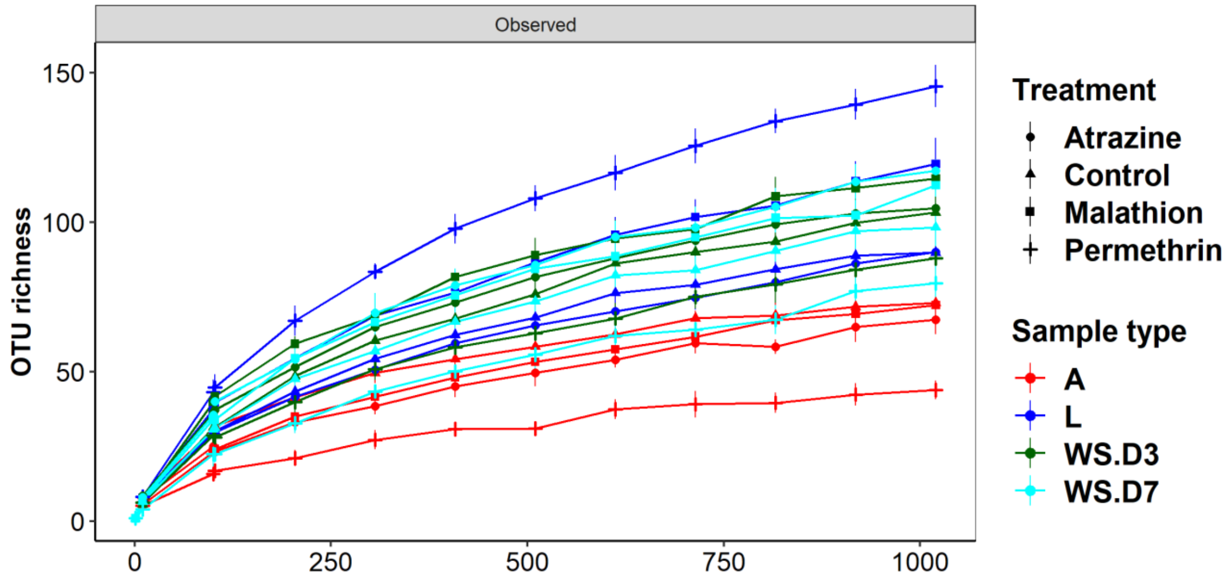

Figure S1. Rarefaction curve analysis of observed richness of bacterial OTUs from samples from *Ae. albopictus* experimental group. WS – Water Sample; L – Larvae; A – Adults; D3 – Day 3; D7 – Day 7. Figures were generated using R version 3.6.1 [77] (https://www.r-project.org/) within the RStudio environment version 1.2.1335 [78] (https://rstudio.com/)

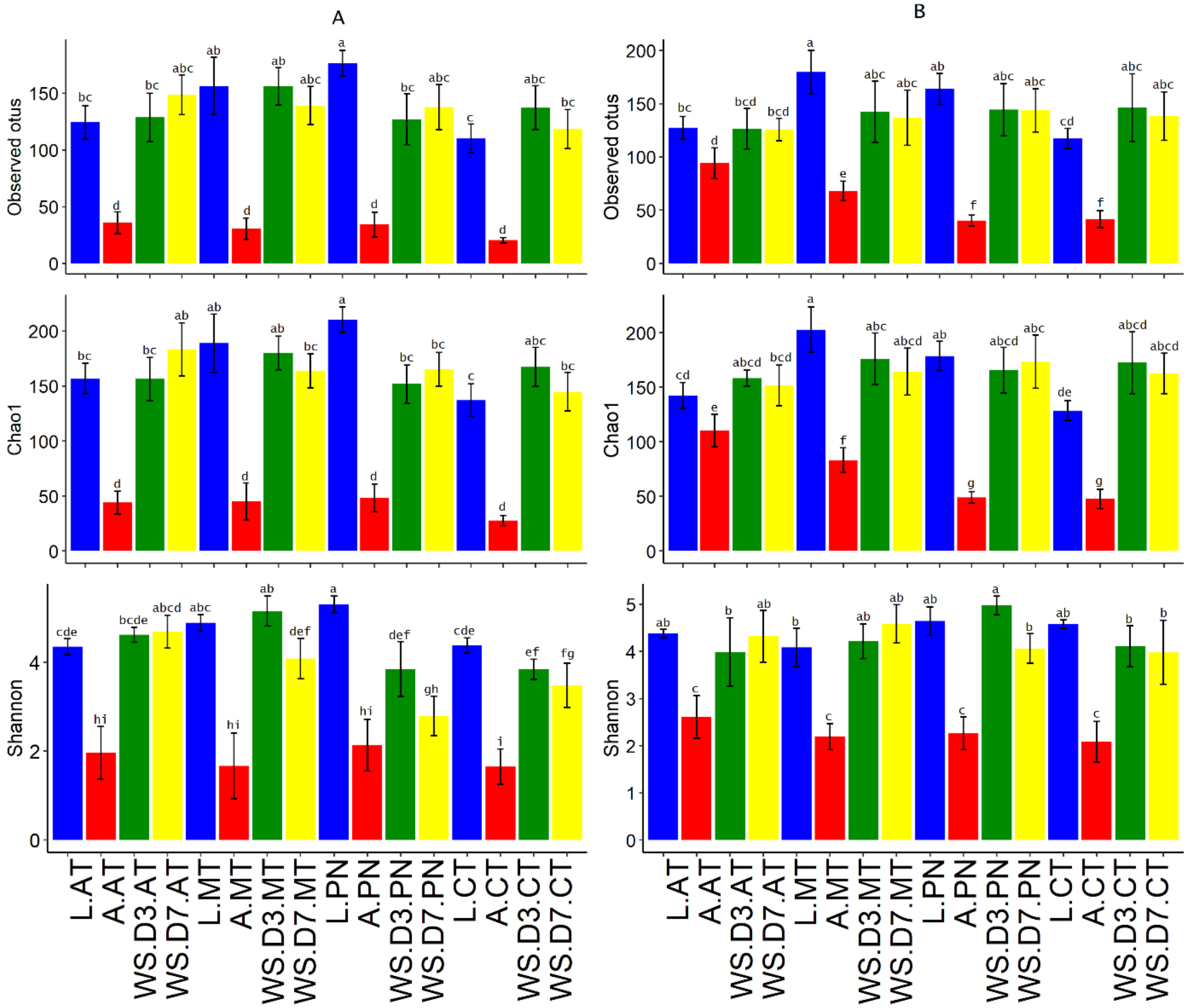

Figure S2. Alpha diversity measures for treatment combinations for *Ae. albopictus* experimental group samples (A), and *Cx. pipiens* L. experimental group samples (B). L – Larvae; A – Adults; AT – Atrazine; MT – Malathion; PN – Permethrin; CT – control; WS – Water sample; D3 – Day 3; D7 – Day 7. (Error bars represent Mean±SE). Figures were generated using R version 3.6.1 [77] (https://www.r-project.org/) within the RStudio environment version 1.2.1335 [78] (https://rstudio.com/)

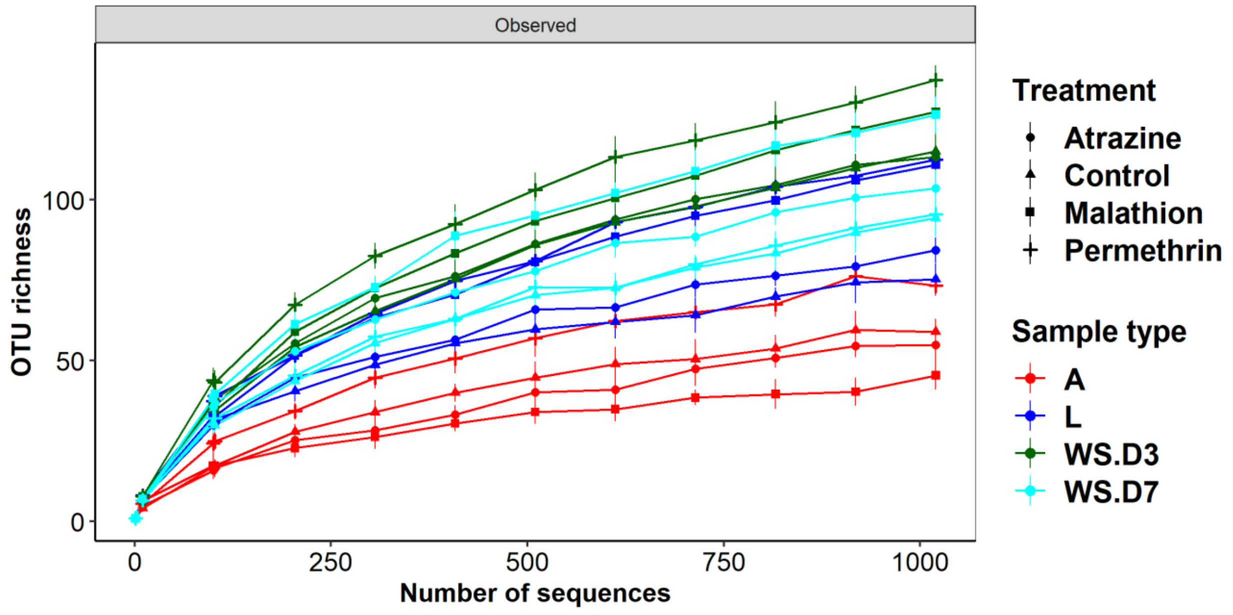

Figure S3. Rarefaction curve analysis of observed richness of bacterial OTUs from samples from *Cx. pipiens* L. experimental group. WS – Water Sample; L – Larvae; A – Adults; D3 – Day 3; D7 – D. Figures were generated using R version 3.6.1 [77] (https://www.r-project.org/) within the RStudio environment version 1.2.1335 [78] (https://rstudio.com/)

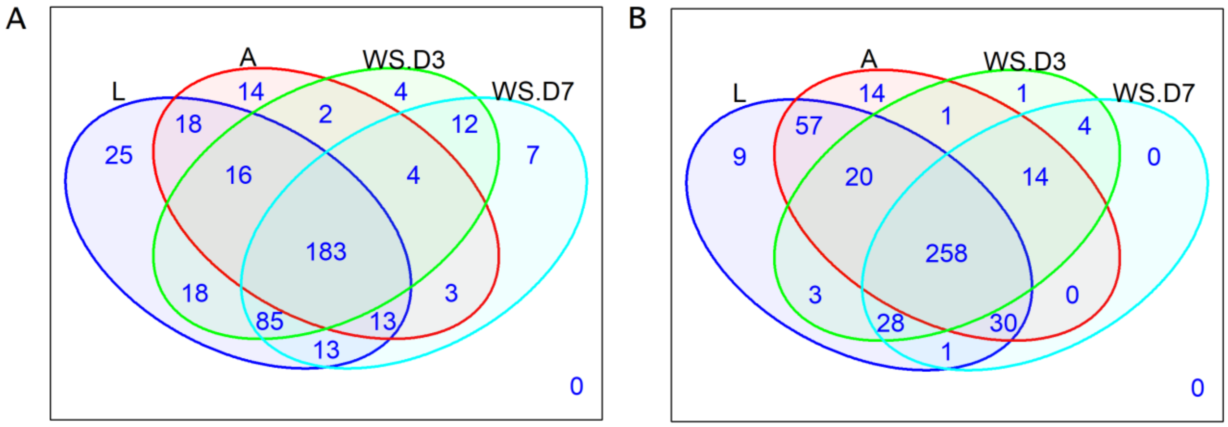

Figure S4. Venn diagrams showing the number of unique and shared bacterial OTUs between mosquito and water samples. Both *Ae. albopictus* and *Cx. pipiens* larval and adult samples from all treatment and control groups were separately pooled together for Venn diagram analysis. **A**: *Ae. albopictus* experimental group; **B**: *Cx. pipiens* L. experimental group. L – Larvae; A – Adults; WS – Water Sample; D3 – Day 3; D7 – Day 7. Figures were generated using R version 3.6.1 [77] (https://www.r-project.org/) within the RStudio environment version 1.2.1335 [78] (https://rstudio.com/)

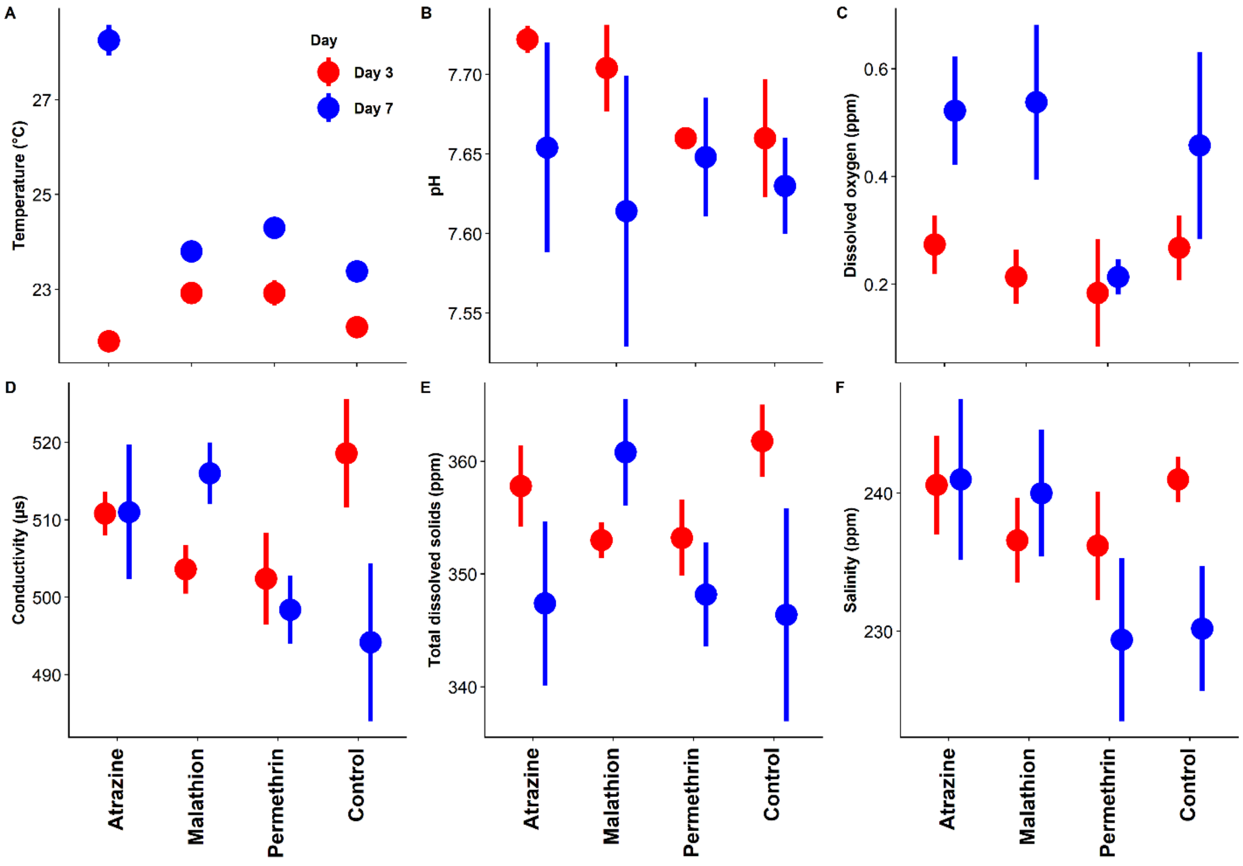

Figure S5. Temporal changes from Day 3 to Day 7 in the physiochemical characteristics of water samples collected from different pesticide treatments for *Ae. albopictus* experimental group (Mean±SE; some error bars are too narrow to be visible). Figures were generated using R version 3.6.1 [77] (https://www.r-project.org/) within the RStudio environment version 1.2.1335 [78] (https://rstudio.com/)

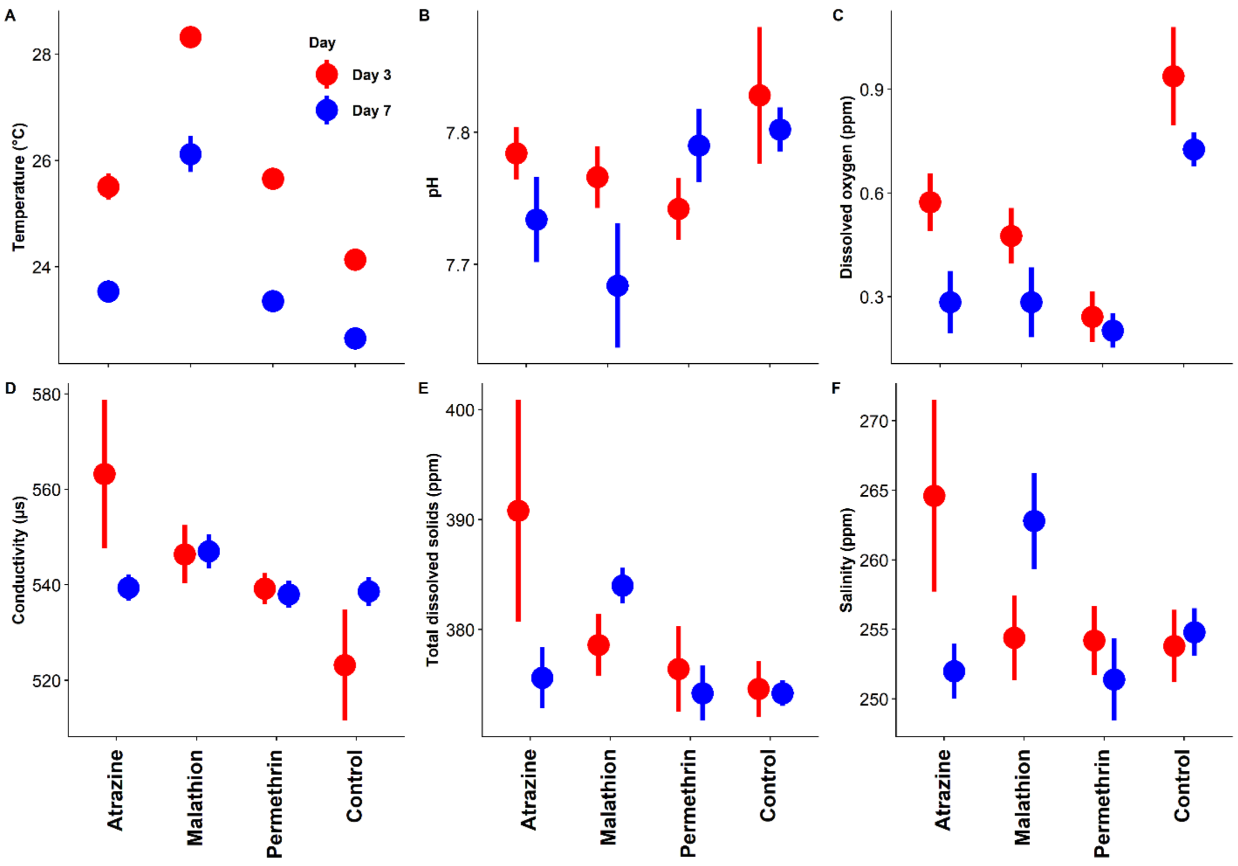

Figure S6. Temporal changes from Day 3 to Day 7 in the physiochemical characteristics of water samples collected from different pesticide treatments for *Cx. pipiens* L. experimental group (Mean±SE; some error bars are too narrow to be visible). Figures were generated using R version 3.6.1 [77] (https://www.r-project.org/) within the RStudio environment version 1.2.1335 [78] (https://rstudio.com/)

Figure S7. Larval mortality rates from the microcosms for (A) *Cx. pipiens* L. and (B) *Ae. albopictus*. Figures were generated using R version 3.6.1 [77] (https://www.r-project.org/) within the RStudio environment version 1.2.1335 [78] (https://rstudio.com/)

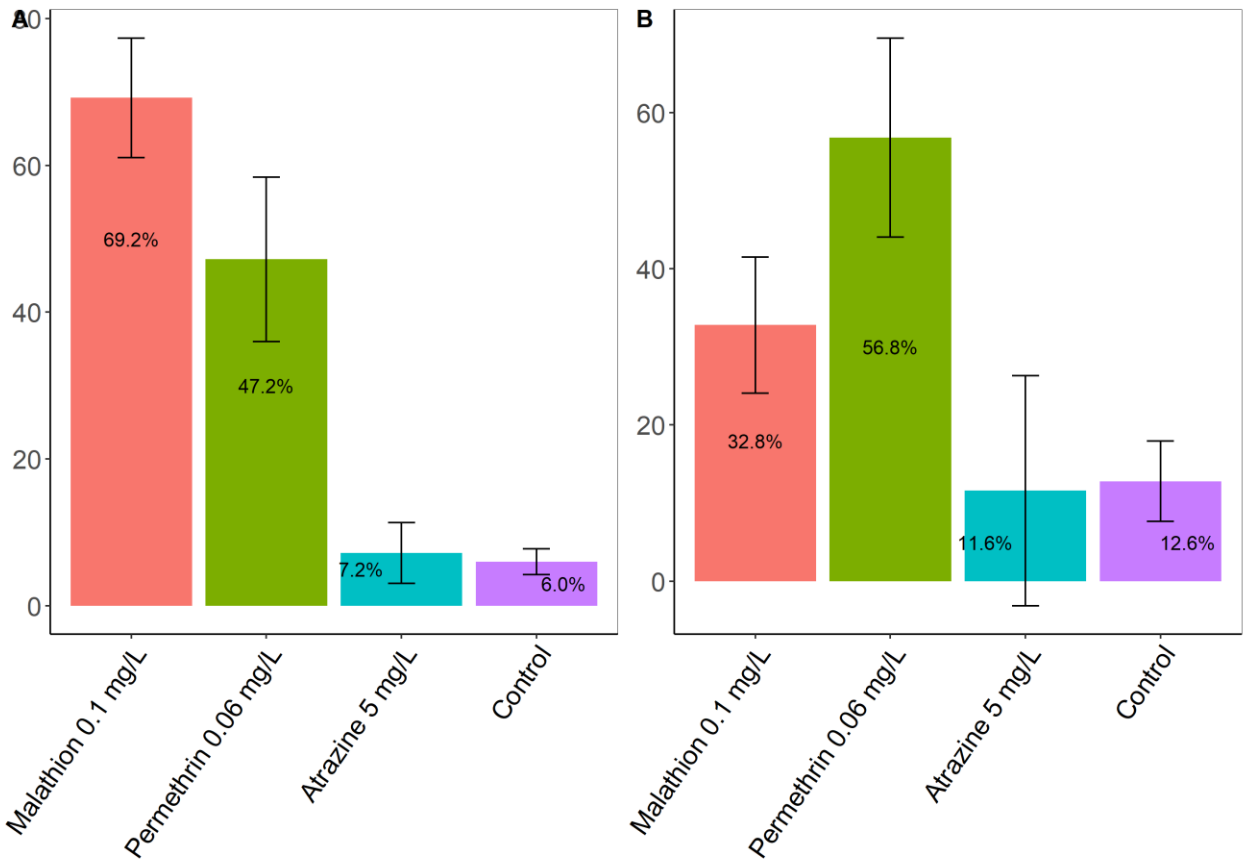

SUPPLEMENTARY TABLES

Table S1. Multivariate non-parametric test of the effect of pesticide and water sampling day on the physical and chemical characteristics of the water samples for *Ae. albopictus* experimental group. The relative effect property measures the probability that an outcome under the experimental treatment is better than an outcome under control

Variable	Wilks' Lambda	df	*P*	Relative effects						
				**Variable**	**pH**	**Temperature**	**Conductivity**	**TDS**	**Salinity**	**Dissolved oxygen**
Pesticide	0.89	18.0, 88.17	0.60	Atrazine	0.63625	0.5225	0.595	0.485	0.60125	0.605
				Malathion	0.49625	0.52125	0.5675	0.5575	0.53375	0.5325
				Permethrin	0.41375	0.5975	0.355	0.4325	0.38125	0.3425
				Control	0.45375	0.35875	0.4825	0.525	0.48375	0.52
Pesticide by sampling day	3.85	42.0, 130.09	<0.0001	Atrazine.day 3	0.7575	0.1075	0.5975	0.605	0.625	0.4675
				Atrazine.day 7	0.515	0.9375	0.5925	0.365	0.5775	0.7425
				Malathion.day 3	0.62	0.36	0.41	0.445	0.495	0.3675
				Malathion.day 7	0.3725	0.6825	0.725	0.67	0.5725	0.6975
				Permethrin.day 3	0.3925	0.39	0.415	0.48	0.4725	0.3175
				Permethrin.day 7	0.435	0.805	0.295	0.385	0.29	0.3675
				Control.day 3	0.5225	0.175	0.6975	0.735	0.65	0.455
				Control.day 7	0.385	0.5425	0.2675	0.315	0.3175	0.585

Table S2. Multivariate non-parametric test of the effect of pesticide and water sampling day on the physical and chemical characteristics of the water samples for *Cx. pipiens* L. experimental group. The relative effect property measures the probability that an outcome under the experimental treatment is better than an outcome under control

Variable	Wilks' Lambda	df	*P*	Relative effects						
				**Variable**	**pH**	**Temperature**	**Conductivity**	**TDS**	**Salinity**	**Dissolved oxygen**
Pesticide	5.60	18.0, 88.17	<0.0001	Atrazine	0.47625	0.4675	0.6175	0.56375	0.53875	0.48125
				Malathion	0.68625	0.25	0.31125	0.37875	0.465	0.8325
				Permethrin	0.36	0.84875	0.6475	0.6675	0.6175	0.43625
				Control	0.4775	0.43375	0.42375	0.39	0.37875	0.25
Pesticide by sampling day	7.12	42.00, 130.09	<0.0001	Atrazine.day 3	0.5925	0.6525	0.7725	0.715	0.705	0.64
				Atrazine.day 7	0.36	0.2825	0.4625	0.4125	0.3725	0.3225
				Malathion.day 3	0.685	0.4375	0.2175	0.395	0.41	0.8575
				Malathion.day 7	0.6875	0.0625	0.405	0.3625	0.52	0.8075
				Permethrin.day 3	0.4775	0.9375	0.59	0.5425	0.4475	0.555
				Permethrin.day 7	0.2425	0.76	0.705	0.7925	0.7875	0.3175
				Control.day 3	0.3925	0.65	0.4525	0.425	0.45	0.265
				Control.day 7	0.5625	0.2175	0.395	0.355	0.3075	0.235
